# Supplementary material for: Robust hemostatic bandages based on nanoclay electrospun membranes
Source: Nat Commun. 2021 Oct 11;12:5922. doi: 10.1038/s41467-021-26237-4 (PMC8505635; doi:10.1038/s41467-021-26237-4)
Supplement: Supplementary file 1 — Supplementary Information [file 41467_2021_26237_MOESM1_ESM.pdf]

Supplementary information

Robust Hemostatic Bandages Based on Nanoclay Electrospun Membranes

Yan Cui[1,2,†], Zongwang Huang[1,†], Li Lei[3,†], Qinglin Li[4,5,†], Jinlong Jiang[6], Qinghai Zeng[3,⋆], Aidong Tang[2,⋆], Huaming Yang[1,⋆] & Yi Zhang[1,⋆ ✉]

[1]Department of Inorganic Materials, School of Minerals Processing and Bioengineering, Central South University, Changsha 410083, China.

[2]College of Chemistry and Chemical Engineering, Central South University, Changsha 410083, China.

[3]Department of Dermatology, the Third Xiangya Hospital, Central South University, Changsha 410013, China.

[4]Cancer Hospital of the University of Chinese Academy of Sciences (Zhejiang Cancer Hospital).

[5]Institute of Cancer and Basic Medicine (IBMC), Chinese Academy of Sciences.

[6]Jiangsu Provincial Key Laboratory of Palygorskite Science and Applied Technology, Huaiyin Institute of Technology, Huaian 223003, P. R. China.

†These authors contributed equally: Yan Cui, Zongwang Huang, Li Lei, and Qinglin Li.

⋆These authors jointly supervised this work: Qinghai Zeng, Aidong Tang, Huaming Yang, Yi Zhang

✉ email: yee_z10@csu.edu.cn

Supplementary Figures

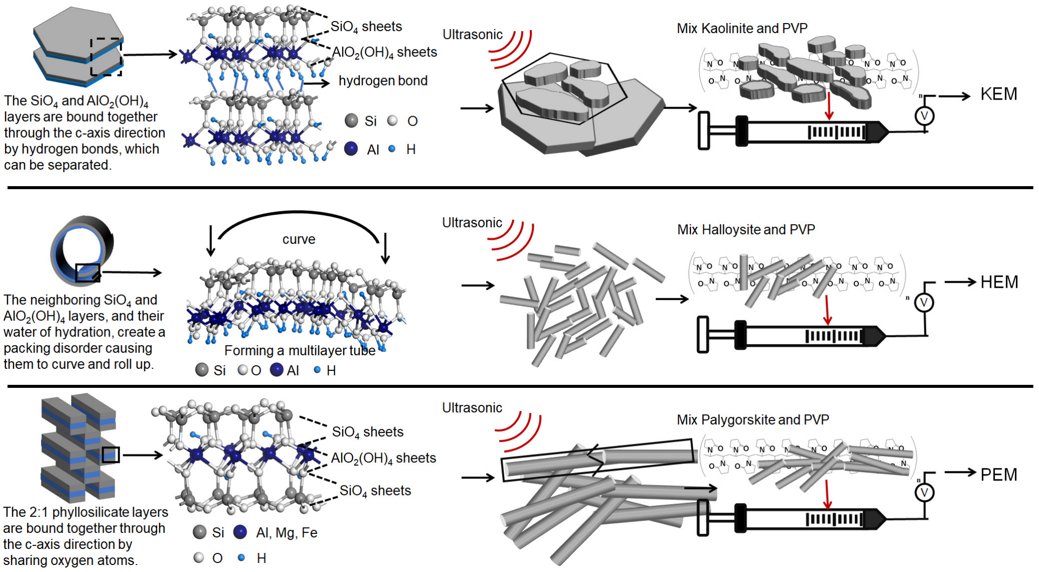

Supplementary Figure 1 Illustration of the preparation route of the KEM, PEM and HEM.

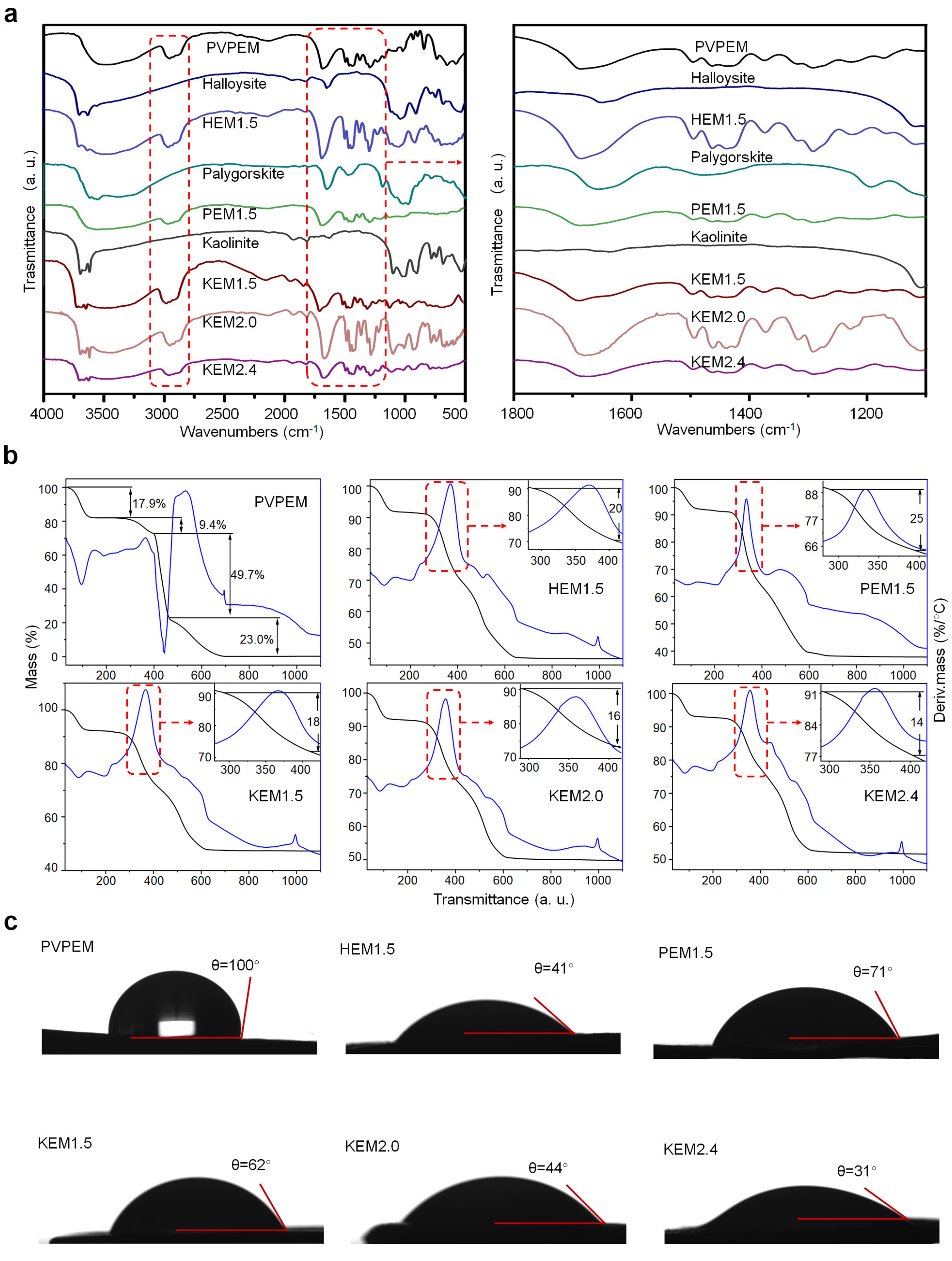

Supplementary Figure 2 (a) The FT-IR spectra of PVPEM, Halloysite, $HEM_{1.5}$, Palygorskite, $PEM_{1.5}$, Kaolinite, $KEM_{1.5}$, $KEM_{2.0}$ and $KEM_{2.4}$, respectively. (b) TG-DSC curves of $HEM_{1.5}$, $PEM_{1.5}$, $KEM_{1.5}$, $KEM_{2.0}$, and $KEM_{2.4}$. (c) Water contact angles for PVPEM, $PEM_{1.5}$, $HEM_{1.5}$, $KEM_{1.5}$, $KEM_{2.0}$ and $KEM_{2.4}$. Source data are provided as a Source Data file.

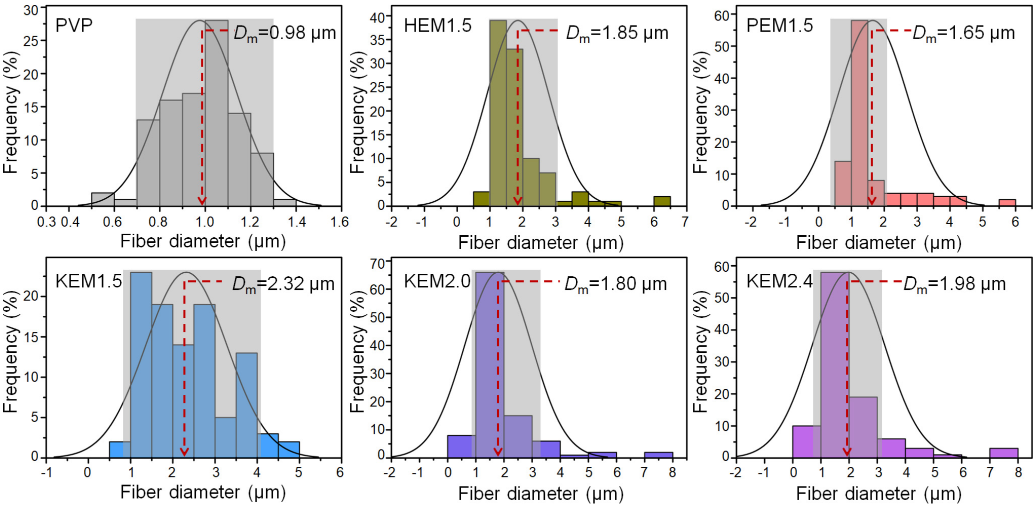

Supplementary Figure 3 The size distributions of the fibers in PVPEM, $PEM_{1.5}$, $HEM_{1.5}$, $KEM_{1.5}$, $KEM_{2.0}$ and $KEM_{2.4}$, respectively, with normalized curves. Source data are provided as a Source Data file.

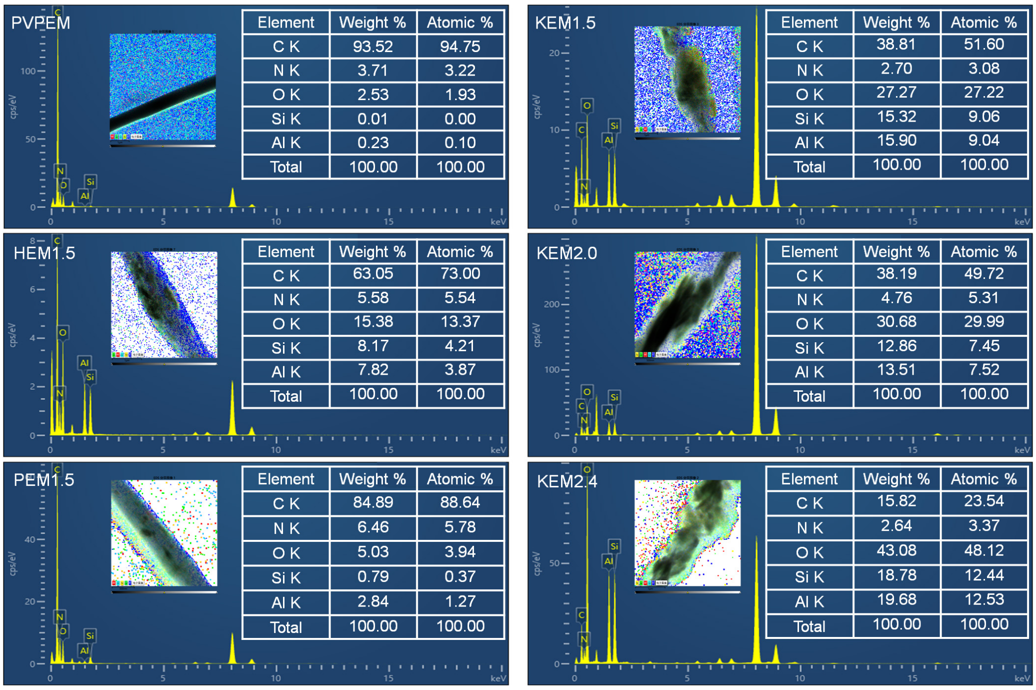

Supplementary Figure 4 The EDX elements analysis for PVPEM, $PEM_{1.5}$, $HEM_{1.5}$, $KEM_{1.5}$, $KEM_{2.0}$ and $KEM_{2.4}$. Source data are provided as a Source Data file.

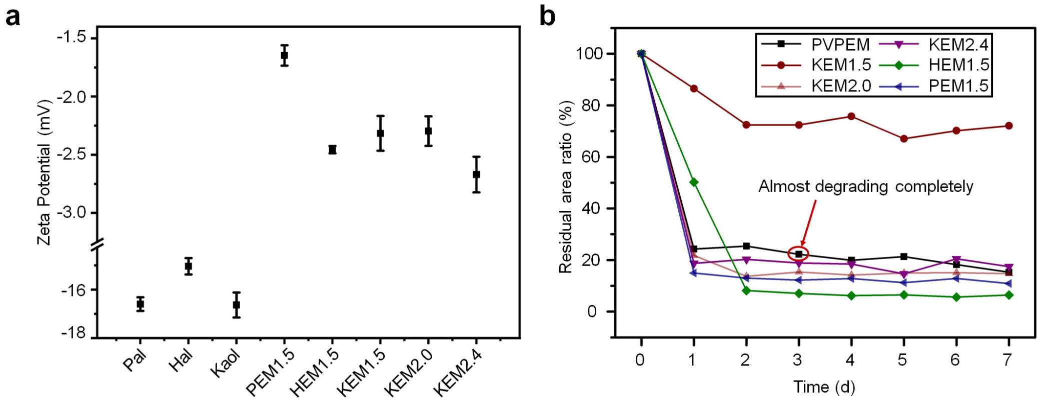

Supplementary Figure 5 Zeta potential for Palygorskite (*n* = 3), Halloysite (*n* = 3), Kaolinite (*n* = 3), $HEM_{1.5}$ (*n* = 3), $PEM_{1.5}$ (*n* = 3), $KEM_{1.5}$ (*n* = 3), $KEM_{2.0}$ (*n* = 3) and $KEM_{2.4}$ (*n* = 3) in neutral solution (similar to the cellular systems). The residual area ratio under continuous 7 days shrinkage test for PVPEM, $HEM_{1.5}$, $PEM_{1.5}$, $KEM_{1.5}$, $KEM_{2.0}$, and $KEM_{2.4}$. Data were analyzed from at least three independently experiments. Source data are provided as a Source Data file.

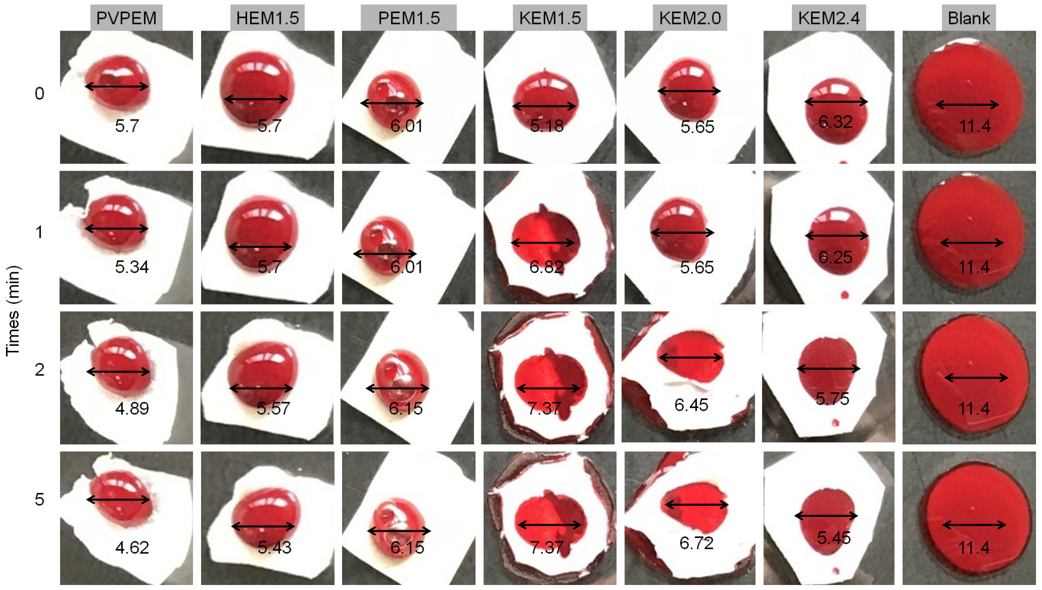

Supplementary Figure 6 Photographs for in vitro blood coagulation experiment of PVPEM, $HEM_{1.5}$, $PEM_{1.5}$, $KEM_{1.5}$, $KEM_{2.0}$, $KEM_{2.4}$ and blank group. Scale units, mm. Data were analyzed from at least three independently experiments. Source data are provided as a Source Data file.

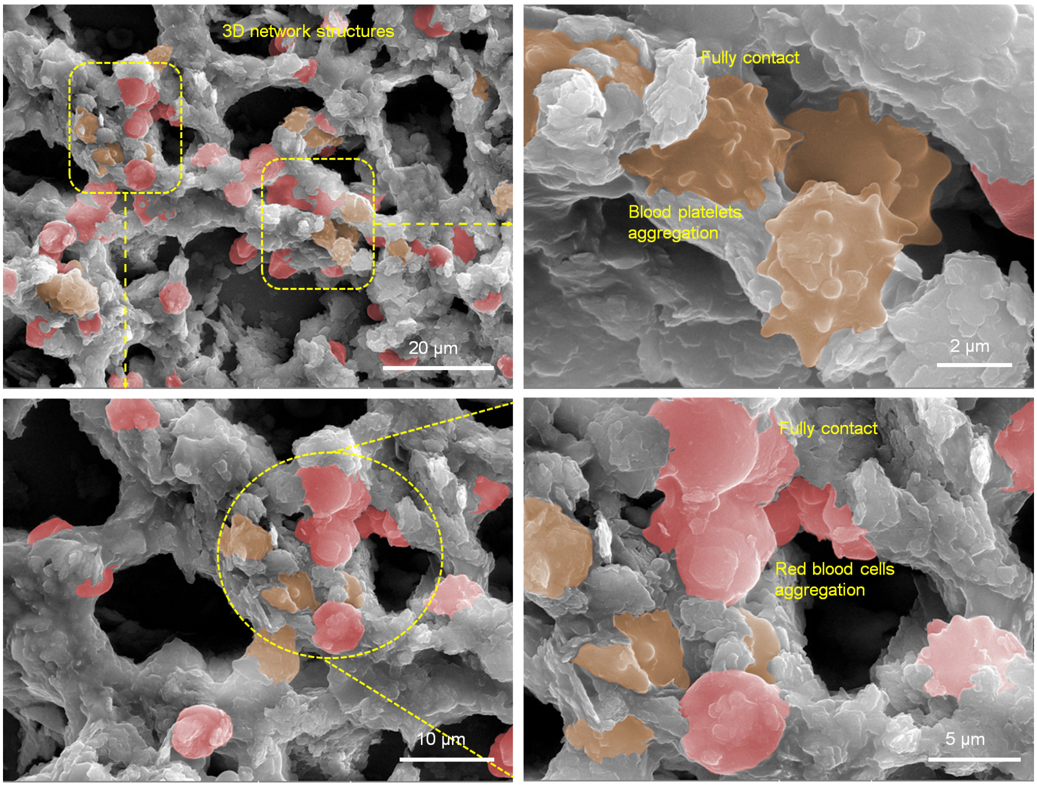

Supplementary Figure 7 The SEM images of blood components interaction with the $KEM_{1.5}$ in the 3D network structures. Red represents red blood cells; Yellow represents the platelets. Data were analyzed from at least three independently experiments. Source data are provided as a Source Data file.

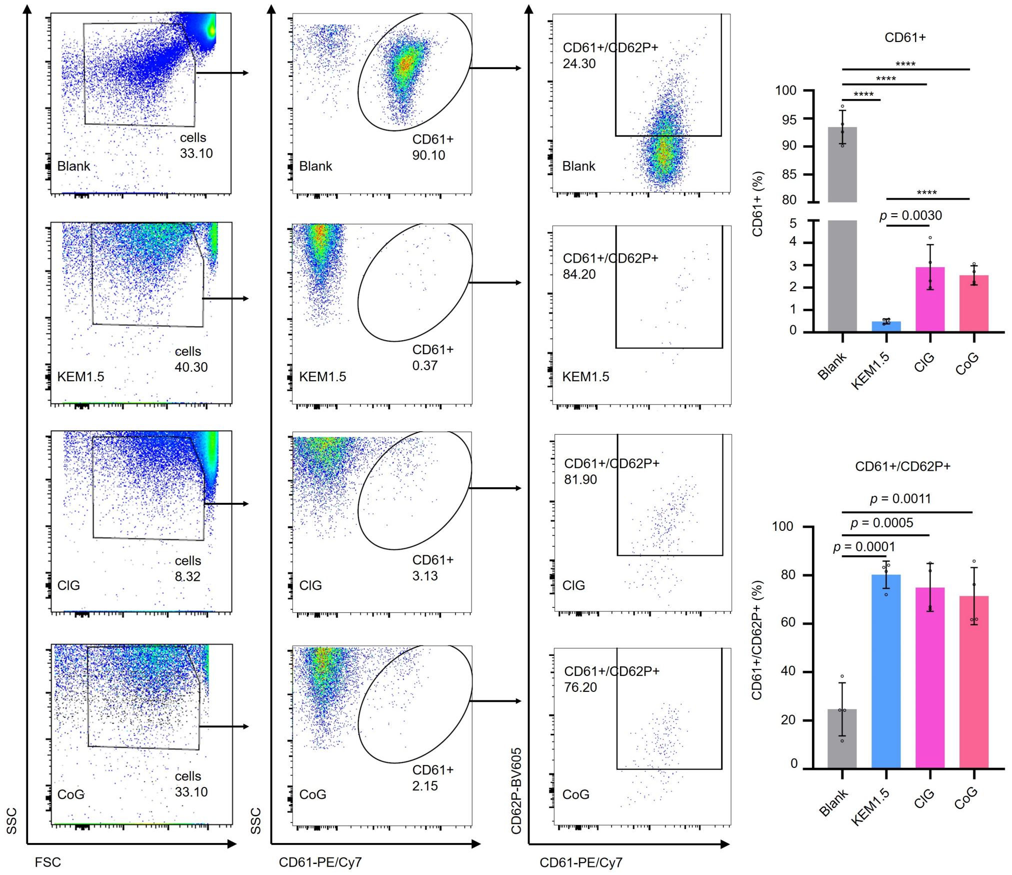

Supplementary Figure 8 Platelet aggregation and activation: The percentage of $CD61^+$ and $CD61^+/CD62P^+$ cells after function with $KEM_{1.5}$, ClG, CoG and without materials (blank group), respectively. $n = 4$ (independent experiments). Data were expressed as mean ± s.d.; ****, $p < 0.0001$. Student's t-test (two-sided) was used for statistical analysis of between two groups comparison. Source data are provided as a Source Data file.

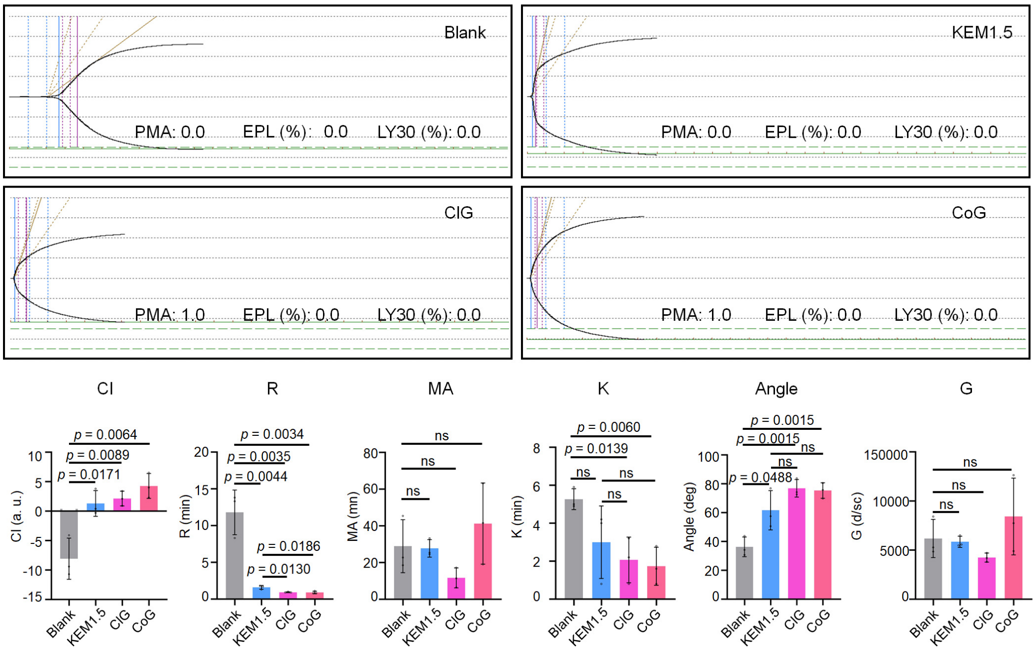

Supplementary Figure 9 The values and traces of Thrombelastograph (TEG) of the whole blood samples after function with $KEM_{1.5}$, ClG, CoG and without materials (blank group), respectively. (CI= Coagulation composite index, R=Reaction time, MA= Maximum amplitude, K=Clot formation time, Angle = α° of the greatest amplitude on the TEG trace, G= Mechanical strength of clot, PMA= Peripheral blood platelets-monocyte aggregates, EPL= Prediction of fibrinolysis index, LY30= Percentage lysis 30 min post-MA.). n=3 (independent experiments). Data were expressed as mean ± s.d.. Student's t-test (two-sided) was used for statistical analysis of between two groups comparison. Source data are provided as a Source Data file.

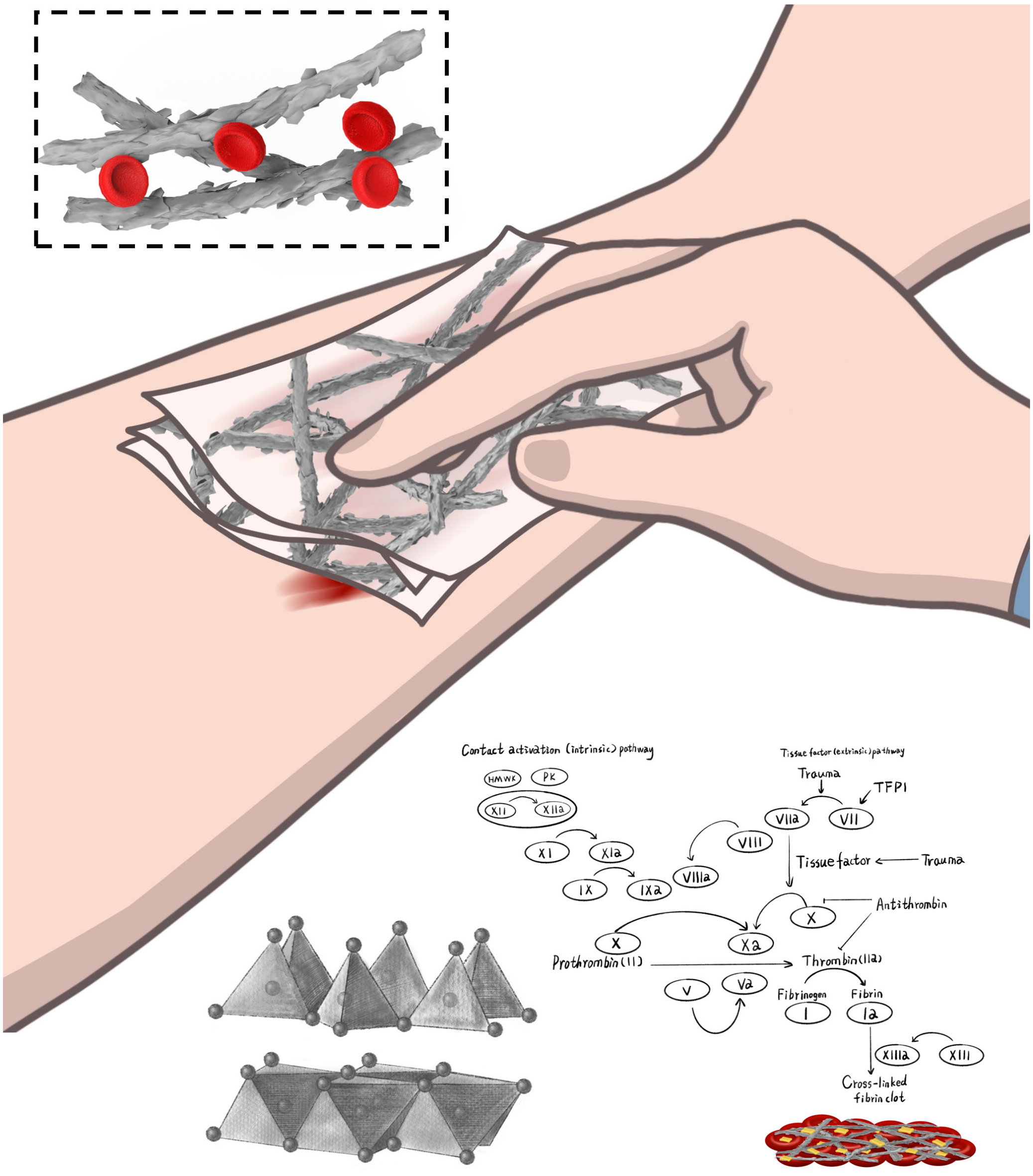

Supplementary Figure 10 Illustration of the intrinsic (contact activation) coagulation cascade pathway activated by KEM in hemostasis.

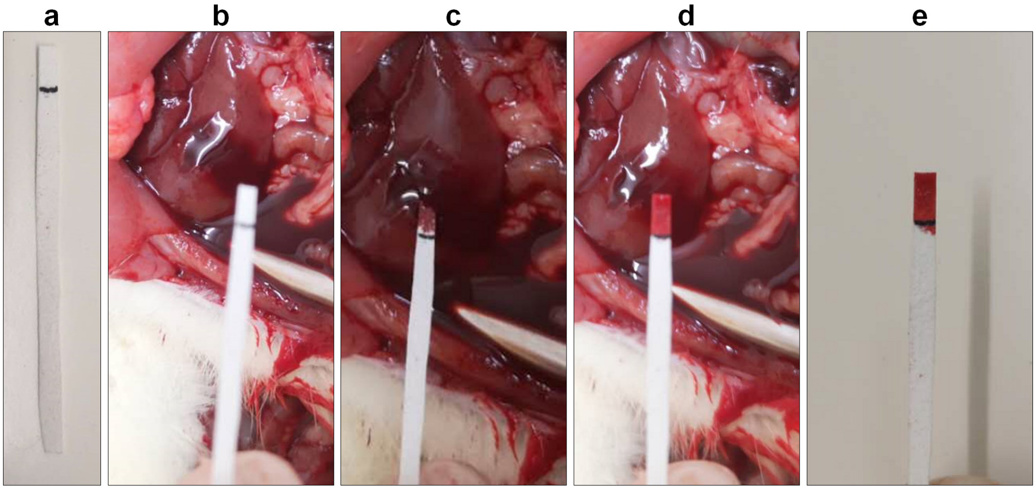

Supplementary Figure 11 The initial bleeding assessment. (a) Using filter paper to make an indicator bar with a width of 0.2 cm, and drawing a black indicator line at 0.5 cm from the end of the indicator bar. (b) After wound bleeding, the indicator bar was immediately place on the wound. (c) Then the blood would seep along the indicator bar. (d) If the blood seep to the black line within 3 seconds, indicating that the bleeding speed and initial amount of bleeding are qualified for subsequent hemostatic experiments. (e) The status of the indicator bar after the initial bleeding assessment was finished.

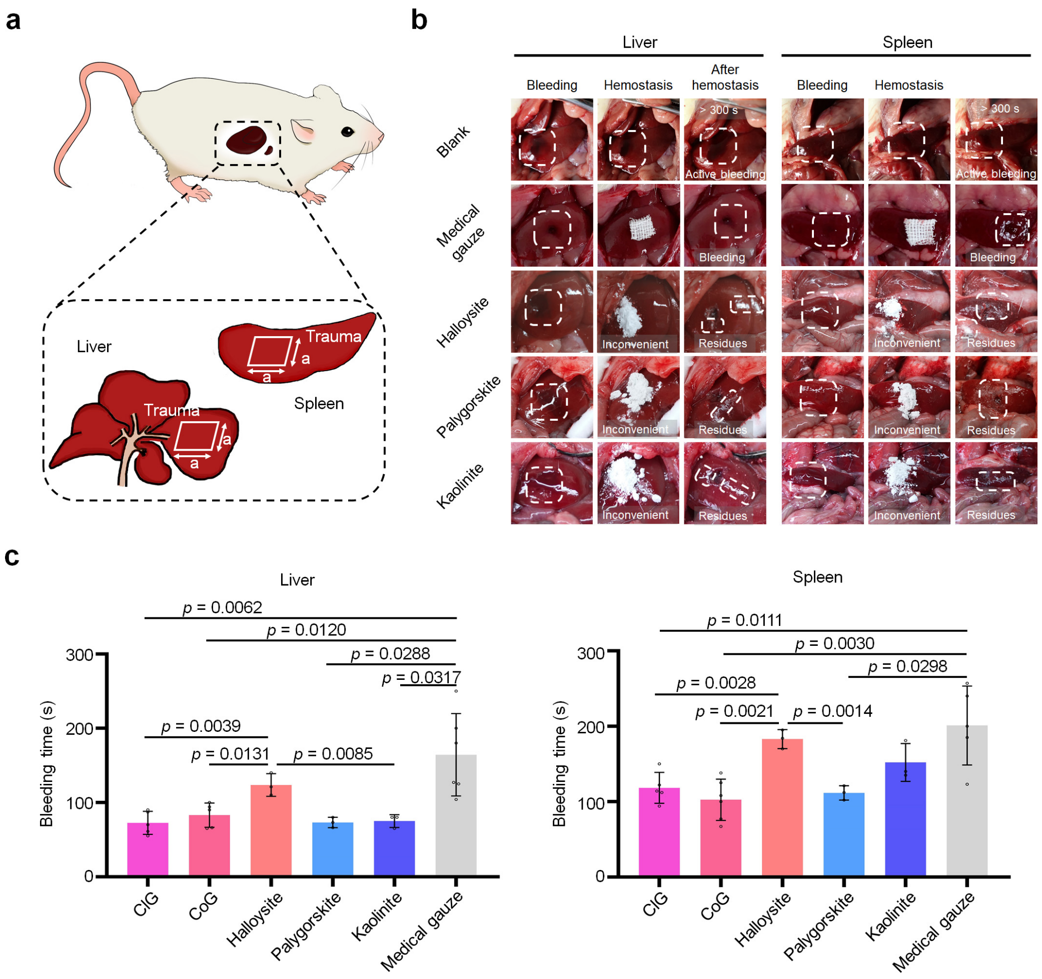

Supplementary Figure 12 (a) Schematic illustrations of rat liver and spleen surface trauma models for NEMs hemostasis experiment. (b) Photographs for the bleeding wound with untreated, treated and removed blank, medical gauze, halloysite, palygorskite, kaolinite group. Blank group hemostasis result didn't present in histogram. (c) Bleeding time of wounds treated with NEMs. In rat liver hemostasis model: ClG (n = 5), CoG (n = 5), halloysite (n = 3), palygorskite (n = 3), kaolinite (n = 3) and medical gauze (n = 6); and in rat spleen hemostasis model: ClG (n = 5), CoG (n = 6), halloysite (n = 3), palygorskite (n = 3), kaolinite (n = 3) and medical gauze (n = 5). Data were expressed as mean ± s.d.. Student's t-test (two-sided) was used for statistical analysis of between two groups comparison in (c). Source data are provided as a Source Data file.

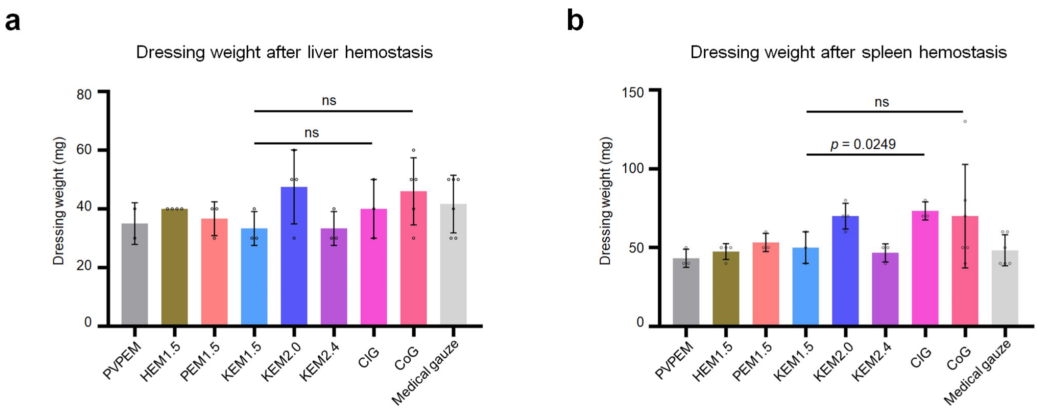

Supplementary Figure 13 (a) The dressing weight after liver hemostasis for PVPEM (n = 2), $HEM_{1.5}$ (n = 4), $PEM_{1.5}$ (n = 3), $KEM_{1.5}$ (n = 3), $KEM_{2.0}$ (n = 4), $KEM_{2.4}$ (n = 3), ClG (n = 3), CoG (n =5) and medical gauze (n = 6). (b) The dressing weight after spleen hemostasis for PVPEM (n = 3), $HEM_{1.5}$ (n = 4), $PEM_{1.5}$ (n = 3), $KEM_{1.5}$ (n = 3), $KEM_{2.0}$ (n = 4), $KEM_{2.4}$ (n = 3), ClG (n = 3), CoG (n = 6) and medical gauze (n = 6). Data were expressed as mean ± s.d.. Student's t-test (two-sided) was used for statistical analysis of between two groups comparison in (a) and (b). Source data are provided as a Source Data file.

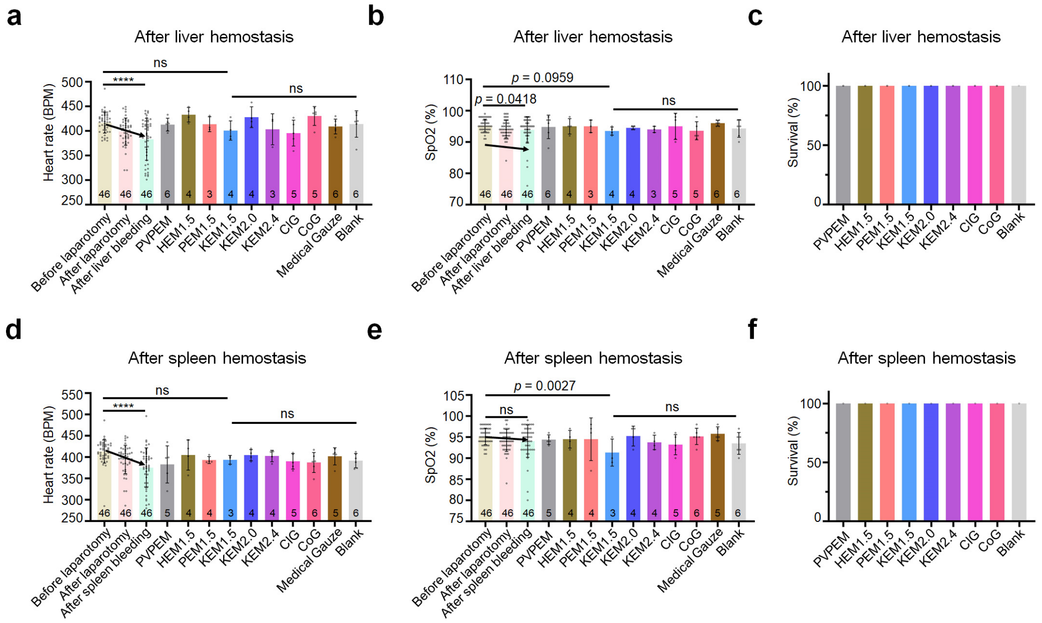

Supplementary Figure 14 (a) The heart rates, (b) blood oxygen saturations, (c) survival rates of PVPEM, HEM$_{1.5}$, PEM$_{1.5}$, KEM$_{1.5}$, KEM$_{2.0}$, KEM$_{2.4}$, ClG, CoG, medical gauze and blank group in rat liver hemostasis model. (d) The heart rates, (e) blood oxygen saturations, (f) survival rates of PVPEM, HEM$_{1.5}$, PEM$_{1.5}$, KEM$_{1.5}$, KEM$_{2.0}$, KEM$_{2.4}$, ClG, CoG, medical gauze and blank group in rat spleen hemostasis model. For heart rate and blood oxygen saturation assessment before laparotomy, after laparotomy and after spleen bleeding, the data of all rats were collected to assess the overall changes. Data were analyzed from more than three experiments and the exact values of n are reported in the Figure. Data were expressed as mean ± s.d.; ****, $p < 0.0001$. Student's t-test (two-sided) was used for statistical analysis of between two groups comparison. Source data are provided as a Source Data file.

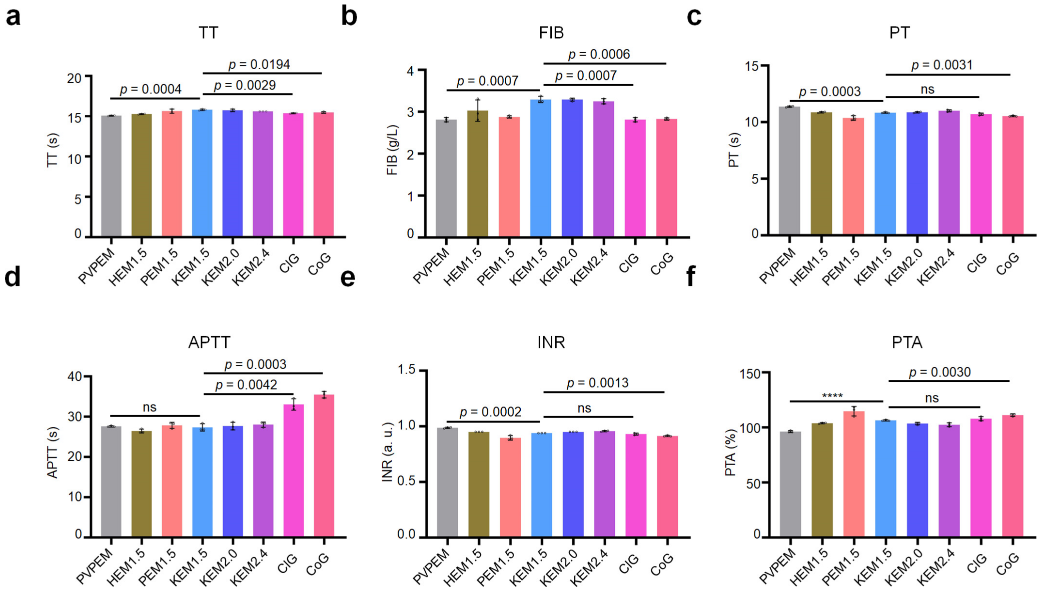

Supplementary Figure 15 (a) The thrombin time (TT), (b) fibrinogen (FIB), (c) prothrombin time (PT), (d) activated partial thromboplastin time (APTT), (e) international Normalized Ratio (INR), (f) prothrombin activity (PTA) for PVPEM, $HEM_{1.5}$, $PEM_{1.5}$, $KEM_{1.5}$, $KEM_{2.0}$, $KEM_{2.4}$ ClG and CoG, respectively. $n = 3$ (independent experiments). Data were expressed as mean ± s.d.; ****, $p < 0.0001$. Student's t-test (two-sided) was used for statistical analysis of between two groups comparison. Source data are provided as a Source Data file.

Supplementary Tables

Supplementary Table 1 The detailed ratios of samples.

Sample	Nanoclay characteristics				Dosage in this study	
	Kinds	Phyllosilicates type	Ideal stoichiometry	Nanoclay Mass ratio (%)	Nanoclay Dosage (g)	PVP Dosage (g)
PVPEM	No	-	$(C_6H_9NO)_n$	0	-	1.0
$HEM_{1.5}$	Halloysite	1:1	$Al_2Si_2O_5(OH)_4$ $\cdot nH_2O$ (n=2 or 4)	60.0	1.5	1.0
$PEM_{1.5}$	Palygorskite	2:1	$Mg_5Si_8O_{20}(OH)_2$ $(OH_2)_4\cdot 4H_2O$	60.0	1.5	1.0
$KEM_{1.5}$	Kaolinite	1:1	Al $[Si_4O_{10}]$ $(OH)_8$	60.0	1.5	1.0
$KEM_{2.0}$	Kaolinite	1:1	Al $[Si_4O_{10}]$ $(OH)_8$	66.7	2.0	1.0
$KEM_{2.4}$	Kaolinite	1:1	Al $[Si_4O_{10}]$ $(OH)_8$	70.6	2.4	1.0

Supplementary Table 2 Representative examples of various materials recently reported for rapid hemostasis applications

Hemostasis materials and the main components		Benefits	Remarks (specific characteristics, applications and limitations)	Reference
**Commercially available products**	**QuikClot (zeolite) and Combat Gauze (kaolinite)**	**High porosity, large specific surface area and good biocompatibility facilitating to the blood coagulation through absorbing plasma components as well as easily for accessibility and applicability**	**Fast hemostatic performance but the exothermicity (for zeolite) and the poor adhesion active component and substrate existing the risk of residual and difficulty for removal from the wounds as well as restriction on internal tissue hemorrhage application**	**--**
**Other uncommerci-alized materials**	**N-Alkyl chitosan nanofiber membrane**	**Activation of coagulation factors and platelets for blood coagulation in vitro**	**Good effects for minor bleeding but the poor mechanical properties and adhesivity unsuitable for severe bleeding**	**1**
	**Self-assembling peptides (RADA16-I)**	**Physical entrapment of blood components in a network of peptide**	**Excellent performance for hemostasis but the complex fabrication process and expensive cost restriction on practical application**	**2**
	**CNT-reinforced glycidyl methacrylate (GMA) functionalized quaternized chitosan (QCSG/CNT)**	**Injectable and conductive shape memory cryogels presents excellent hemostatic performance in rabbit liver defect lethal noncompressible hemorrhage model (Time: ~3.9 min)**	**Suitable for irregularly shaped hemorrhage but the residual CNT existing the risk of thrombus and difficulty for removal from wound after bleeding-stop**	**3**
	**Mesoporous silica nanoparticles (MSN)**	**Promoting the blood proteins to contact the huge interior surfaces of MSN and then initiate the quick blood clot**	**Accessibility and diffusion of clotting-promoting proteins for hemostasis but the fine MSN particles existing the risk of thrombus and histogenic immunity**	**4**
	**Mesoporous zeolite-cotton hybrid hemostat (mCHA-C)**	**Mesoporous zeolite onto the surface of cotton fiber by on-site template-free growth with higher procoagulant activity and minimized loss of active components in rabbit lethal femoral artery injury model (Time: 159 ± 12 s)**	**Great effects for severe hemorrhage but the complicated fabrication process restricted practical application and existing risk of secondary damage on the wound from exothermicity**	**5**
	**Gelatin/PAAm/Laponite Nanocomposite Hydrogels**	**Hierarchical structures, high transparencies, good homogeneity and ultrastrong tensibilities**	**Less toxic effects but restriction on severe hemolysis, poor mechanical strengh and long clotting time**	**6**
	**Graphene-montmorillonite or graphene-kaolin composite sponge (GMCS)**	**Quickly absorbing plasma to increase the concentration of hemocytes and platelets in rabbit artery injury test (Time: ~85 s)**	**Good biocompatibility but the exfoliated and residual MMT or kaolin particles difficulty for removal and degradation from bleeding tissue site**	**7,8**
**This work**	**Integrated Nanoclay Electrospun Membranes (best: $KEM_{1.5}$)**	**Accelerate the coagulation of blood through rapidly absorbing plasma and aggregating blood components with a highly hydrophilic framework, and FXII could be activated by kaolinite on the integrated fiber surface to trigger the intrinsic coagulation cascade**	**The NEMs with the integrated structure and robust framework have excellent hemostatic capability with low biotoxicity**	**--**

Supplementary Discussion

Thermal stability and interaction of NEMs. Four steps of the DSC-TG curve (Supplementary Fig. 2b) of PVPEM at approximately 30 to 130 °C, 280 to 400 °C, 400 to 465 °C and 465 to 687 °C are assigned to the release of water and solvent, combustion heat release of uncombined PVP (second step), carbonization of PVP, and carbon combustion heat release, respectively[9]. During the second step of the DSC-TG curve of the nanoclay electrospun membranes, the contents of uncombined PVP of HEM (20 %) and PEM (25 %) are higher than that detected in the case of KEM. This phenomenon is similar to the shift observed in the FT-IR spectra. This phenomenon can be explained by the influence of the number of hydroxyl groups in the clay on the content of the uncombined PVP. The kaolinite-based electrospun membranes have the highest hydroxyl content, and the content of uncombined PVP was approximately 14-18% (an increase in the amount of kaolinite further reduced the amount of uncombined PVP), indicating that kaolinite has excellent interaction with the PVP fiber. Nevertheless, the TG curve of the nanoclay electrospun membranes is decreasing more drastically (by over 14%) than that of PVPEM (9.4%) in the second stage, while the PVP content of nanoclay electrospun membranes is less than that of PVPEM. This phenomenon may be attributed to the severe shrinkage of the pure PVP fiber leading to a structural blockage of PVPEM; the uncombined PVP inside the membrane is difficult to burn at the temperature of the second step. In the third step, the PVPEM curve shows a large decrease (considerably larger than that observed in the case of the nanoclay electrospun membranes), indicating that the majority of uncombined PVP in PVPEM is burned during the third step. Thus, the KEM showed better thermal stability than that of HEM, PEM and PVPEM due to strong interaction with PVP[10,11].

The hydrophilicity and fiber size of NEMs. The static water-contact angles show great decrease from PVPEM (100°) to the NEMs (< 71°), indicating the improvement of hydrophilicity due to incorporation of nanoclays (Supplementary Fig. 2c). The KEM showed an obvious improvement of hydrophilicity (contact angle from 62° to 31°) with the increase additive mass ratio of kaolinite (from 60 % to 70.6 %), indicating that hydrophilicity was related to the clay mass ratio in NEMs. In Supplementary Fig. 3, the NEM fiber size distribution results showed monomodal distribution curves, which were consistent with the SEM images. The NEM fibers (> 1.65 μm) showed a larger size than that of PVPEM (0.98 μm) due to the nanoclay incorporated and guided growth, indicating a higher elasticity modulus and strain peak stress that improved the mechanical strength. The diameter of $KEM_{1.5}$ (2.32 μm) was more evenly and larger than other NEMs (Supplementary Fig. 3), which may be attributed to the particle agglomerates causing additional gravity stretching that led to fiber elongation and diameter decrease in other NEMs[12,13].

The composition and shrinkage of NEMs. In the energy dispersive X-Ray spectroscopy (EDX) analysis (Supplementary Fig. 4), the pure PVP nanofibers were composed of C, N and O elements and almost no Si (wight: 0.01%) and Al (wight: 0.23%) elements. Importantly, the outer layer of HEM, PEM and KEM nanofibers were composed of C, N and O elements, while there are also obvious Si and Al elements distribution in the nanofibers (*e.g.* wight: 15.32 % and 15.90 % for Si and Al in $KEM_{1.5}$), indicating the incorporation of nanoclay and indirectly verifying the clay particles dispersion. The shrinkage experiment for continuous 7 days was used to further characterize the interaction in the nanoclay-organic frameworks. The effective area (< 30% shrinkage ratio) was well retained by the uniformly dispersed kaolinite in $KEM_{1.5}$. The pure PVPEM showed an obvious shrinkage at room temperature (> 75% shrinkage ratio)[10,11] and was

almost degraded completely (only a thin layer remained) after 3 days (in direct observation result), which restricts the practical applicability and increases the costs. The other NEMs shrunk (rolling up or folding) after 2 days (in direct observation result) causing a similar area decrease to PVPEM, which may be attributed to the nanoclay agglomerates and poor dispersion.

The hemostatic capability and biocompatibility. The photograph showed that the blood could not absorbed and clot in PVPEM, $PEM_{1.5}$, and $HEM_{1.5}$ within 5 min; however, the membranes showed a small wrinkle due to formation of the intermolecular hydrogen bonds with PVP by the water molecules of the blood (Supplementary Fig. 6). The blood infiltrated into $KEM_{1.5}$ and $KEM_{2.0}$ ($KEM_{2.4}$) within one and two minutes and coagulated within two and five minutes, respectively, benefiting from the hydrophilicity and easy contact with the active particles on KEM surface. In blank group, the blood (with the action of EDTA) was clot after 60 min, indicating that the clotting time is shortened by 12-30-fold in KEM (best: $KEM_{1.5}$). The active bleeding would not stop over 300 s in blank groups of both spleen and liver wound (the results didn't present in the histogram of Supplementary Fig. 12), and the common medical gauze (the control group of just 'compression') showed a long hemostatic time (liver: 164 ± 56 s, spleen: 201 ± 52 s), which may be due to without any hemostatic active components (Supplementary Fig. 12). There were numerous residues of the three hemostatic powders on the wound that necessitate scrupulous debridement and has a high risk of distal thrombosis and inflammation[5].

The changes in rat heart rate and blood oxygen saturation (SpO_2) were evaluated during the hemostatic process of each material (Supplementary Fig. 14). The rat heart rate showed a significant difference ($P<0.0001$) before and after laparotomy and trauma treatments, which was attributed to the destabilization caused by bleeding (Supplementary Fig. 14). Under continuous

observation to hemostasis completement, all NEMs treatment groups (including ClG and CoG) exhibited a rapid rescue and stabilization for the destabilized rat heart rate accompanied by stable blood oxygen saturation (SpO_2). For the blank group, the wound was given compression to stop the bleeding after 5 minutes due to severe destabilization of heart rate and SpO_2 and unceasing active bleeding that may be related to no active hemostatic components. Although in the liver hemorrhage model, there was a statistical difference in SpO_2 between before laparotomy and $KEM_{1.5}$ treatment, the difference is very small and does not have actual clinical significance. It emphasized that there were no significant differences between $KEM_{1.5}$ and comparison groups on the heart rate and SpO_2, which was consistent with the results in bleeding time and 'relative OD' value, indicating that $KEM_{1.5}$ reaches the 'standard of care' level for commercial products (ClG and CoG)[2,5]. After the hemostasis experiment was done (including an addition compression to stop the bleeding after 5 minutes in blank group), the abdominal cavity was closed and a same (100%) survival of rats was retained over 5 weeks observation in each group (Supplementary Fig. 14).

Furthermore, coagulation related tests were investigated to explore the effect of NEMs on clotting cascade, concerning the safety of coagulation system. The TT, FIB, PT and APTT of all the NEMs, ClG and CoG were within 15.07-17.33 s, 2.81-3.29 g/L, 8.47-11.37 s and 25.17-35.46 s (including INR (0.88-0.99) and PTA (95.7-118.2%)), respectively (Supplementary Fig. 15). Although those results showed slight differences between some groups, the results of TT, FIB, PT, or APTT in all groups were all among normal ranges[14]. It also means that a general level was maintained on TT, FIB, PT, or APTT for all NEMs, ClG and CoG, suggesting a good safety of coagulation system[1,5].

References

1. Wang, X. et al. Exploration of blood coagulation of N-Alkyl chitosan nanofiber membrane in vitro. *Biomacromolecules.* **19**, 731-739 (2018).

2. Hsu, B. B. et al. Clotting mimicry from robust hemostatic bandages based on self-assembling peptides. *ACS Nano* **9**, 9394-9406 (2015).

3. Zhao, X., Guo, B., Wu, H., Liang, Y. & Ma, P. X. Injectable antibacterial conductive nanocomposite cryogels with rapid shape recovery for noncompressible hemorrhage and wound healing. *Nat. Commum.* **9**, 2784 (2018).

4. Chen, Z. et al. Blood clot initiation by mesoporous silica nanoparticles: dependence on pore size or particle size. *J. Mater. Chem. B* **1**, 1-10 (2013).

5. Yu, L., Shang, X., Chen, H., Xiao, L., Zhu, Y. & Fan, J. A tightly-bonded and flexible mesoporous zeolite-cotton hybrid hemostat. *Nat. Commum.* **9**, 09849 (2019).

6. Li, C., Mu, C., Lin, W. & Ngai, T. Gelatin effects on the physicochemical and hemocompatible properties of gelatin/PAAm/laponite nanocomposite hydrogels. *ACS Appl. Mater. Interfaces* **7**, 18732-18741 (2015).

7. Li, G. et al. Graphene-montmorillonite composite sponge for safe and effective hemostasis. *ACS Appl. Mater. Inter.* **8**, 35071-35080 (2016).

8. Liang, Y., Xua, C., Lia, G., Liub, T., Liangb, J. F. & Wang X. Graphene-kaolin composite sponge for rapid and riskless hemostasis. *Colloid Surface B* **169**, 168-175 (2018).

9. Shahhosseininia, M., Bazgir, S. & Joupari, M. D. Fabrication and investigation of silica nanofibers via electrospinning. *Mater Sci Eng C Mater Biol Appl* **91**, 502-511 (2018).

10. Grandgeorge, P., Krins, N., Hourlier-Fargette, A., Laberty-Robert, C., Neukirch, S. & Antkowiak, A. Capillarity-induced folds fuel extreme shape changes in thin wicked membranes. *Science* **360**, 296-299 (2018).

11. Jin,Y. et al. Buckled Au@PVP nanofiber networks for highly transparent and stretchable conductors. *Adv. Electron. Mater.* **2**, 1500302 (2016).

12. McManus, M. C. et al. Mechanical properties of electrospun fibrinogen structures. *Acta Biomater* **2**, 19-28 (2006).

13. Huang, Z. et al. Electrospinning with a spindle-knot structure for effective PM2.5 capture. *Sci China Mater* **64**, 1278-1290 (**2020)**

14. Long, M. et al. Emerging nanoclay composite for effective hemostasis. *Adv. Funct. Mater.* **28**, 1704452 (2018).
