# Supplementary material for: Robust hemostatic bandages based on nanoclay electrospun membranes
Source: Nat Commun. 2021 Oct 11;12:5922. doi: 10.1038/s41467-021-26237-4 (PMC8505635; doi:10.1038/s41467-021-26237-4)
Supplement: Supplementary file 3 — Reporting Summary [file 41467_2021_26237_MOESM3_ESM.pdf]

nature research

Corresponding author(s): Qinghai Zeng, Aidong Tang, Huaming Yang, Yi Zhang

Last updated by author(s): Aug 29, 2021

# Reporting Summary

Nature Research wishes to improve the reproducibility of the work that we publish. This form provides structure for consistency and transparency in reporting. For further information on Nature Research policies, see our Editorial Policies and the Editorial Policy Checklist.

## Statistics

For all statistical analyses, confirm that the following items are present in the figure legend, table legend, main text, or Methods section.

| n/a | Confirmed | |
|---|---|---|
| ☐ | ☒ | The exact sample size (*n*) for each experimental group/condition, given as a discrete number and unit of measurement |
| ☐ | ☒ | A statement on whether measurements were taken from distinct samples or whether the same sample was measured repeatedly |
| ☐ | ☒ | The statistical test(s) used AND whether they are one- or two-sided<br>*Only common tests should be described solely by name; describe more complex techniques in the Methods section.* |
| ☒ | ☐ | A description of all covariates tested |
| ☒ | ☐ | A description of any assumptions or corrections, such as tests of normality and adjustment for multiple comparisons |
| ☐ | ☒ | A full description of the statistical parameters including central tendency (e.g. means) or other basic estimates (e.g. regression coefficient) AND variation (e.g. standard deviation) or associated estimates of uncertainty (e.g. confidence intervals) |
| ☐ | ☒ | For null hypothesis testing, the test statistic (e.g. *F*, *t*, *r*) with confidence intervals, effect sizes, degrees of freedom and *P* value noted<br>*Give P values as exact values whenever suitable.* |
| ☒ | ☐ | For Bayesian analysis, information on the choice of priors and Markov chain Monte Carlo settings |
| ☒ | ☐ | For hierarchical and complex designs, identification of the appropriate level for tests and full reporting of outcomes |
| ☒ | ☐ | Estimates of effect sizes (e.g. Cohen's *d*, Pearson's *r*), indicating how they were calculated |

*Our web collection on statistics for biologists contains articles on many of the points above.*

## Software and code

Policy information about availability of computer code

| | |
|---|---|
| Data collection | Microsoft Excel 2010 was used to data collection. |
| Data analysis | OriginPro (version 8.5) is used for materials properties analysis. GraphPad Prism (version 8.0) is used for the statistical analysis. Flowjo (version 10) is used for flow cytometry analysis. Microsoft PowerPoint 2010 is used for images combination. |

For manuscripts utilizing custom algorithms or software that are central to the research but not yet described in published literature, software must be made available to editors and reviewers. We strongly encourage code deposition in a community repository (e.g. GitHub). See the Nature Research guidelines for submitting code & software for further information.

## Data

Policy information about availability of data

All manuscripts must include a data availability statement. This statement should provide the following information, where applicable:

- Accession codes, unique identifiers, or web links for publicly available datasets
- A list of figures that have associated raw data
- A description of any restrictions on data availability

All data are available within the Article and Supplementary Files, or available from the corresponding authors upon reasonable request. Source data are provided with this paper.

# Field-specific reporting

Please select the one below that is the best fit for your research. If you are not sure, read the appropriate sections before making your selection.

- [x] Life sciences
- [ ] Behavioural & social sciences
- [ ] Ecological, evolutionary & environmental sciences

For a reference copy of the document with all sections, see nature.com/documents/nr-reporting-summary-flat.pdf

# Life sciences study design

All studies must disclose on these points even when the disclosure is negative.

| | |
|---|---|
| Sample size | No sample-size calculation was performed. Sample sizes were determined based on the principle of the number of independent experiments can provide necessary statistical support. All animal experiments were repeated and represented a minimum of three times independently with similar results. |
| Data exclusions | No data were excluded in this study. |
| Replication | All animal experiments were repeated at least three times independently with similar results, including in vitro and in vivo hemostatic tests and all other measurements. All replications showed reproducibly similar data and were successful. |
| Randomization | All SD rats were evenly and randomly divided into different groups for hemostatic performance evaluations (including in vivo rat tail amputation model test, a rat liver and spleen hemostasis model test and other related measurements). All other experiments in this study were also allocated randomly and performed. |
| Blinding | All the experiments were performed by specific operators, who were blinded to group allocation during data collection and analysis. |

# Reporting for specific materials, systems and methods

We require information from authors about some types of materials, experimental systems and methods used in many studies. Here, indicate whether each material, system or method listed is relevant to your study. If you are not sure if a list item applies to your research, read the appropriate section before selecting a response.

## Materials & experimental systems

| n/a | Involved in the study | |
|---|---|---|
| [ ] | [x] | Antibodies |
| [x] | [ ] | Eukaryotic cell lines |
| [x] | [ ] | Palaeontology and archaeology |
| [ ] | [x] | Animals and other organisms |
| [x] | [ ] | Human research participants |
| [x] | [ ] | Clinical data |
| [x] | [ ] | Dual use research of concern |

## Methods

| n/a | Involved in the study | |
|---|---|---|
| [x] | [ ] | ChIP-seq |
| [ ] | [x] | Flow cytometry |
| [x] | [ ] | MRI-based neuroimaging |

## Antibodies

| | |
|---|---|
| Antibodies used | Antibody CD61-PE/Cyanine7 (CD61-PE/Cy7; clone: VI-PL2; catalog: 336416; lot: B318046) and CD62P-Brilliant Violet 605TM (CD62P-BV605; clone: AK4; catalog: 304920; lot: B317810) were purchased from commercial sources (Biolegend, USA). |
| Validation | All antibodies were purchased from commercial sources and were validated by the manufacturer (Biolegend). The website links were as follows:<br>CD61-PE/Cy7 (https://www.biolegend.com/en-us/products/pe-cyanine7-anti-human-cd61-antibody-12732)<br>CD62P-BV605 (https://www.biolegend.com/en-us/products/brilliant-violet-605-anti-human-cd62p-p-selectin-antibody-9623) |

## Animals and other organisms

Policy information about studies involving animals; ARRIVE guidelines recommended for reporting animal research

| | |
|---|---|
| Laboratory animals | All male Sprague Dawley (SD) rats were obtained (220-250 g, 7-8 weeks) from the Hunan SJA Laboratory Animal Co., Ltd (Hunan, China). |
| Wild animals | This study doesn't involve wild animals. |
| Field-collected samples | This study doesn't involve samples collected from the field. |

| | |
|---|---|
| Ethics oversight | All of the animal experiments were performed according to the relevant ethical regulations of Central South University, and this study received approval from Central South University Experimental Animal Ethics Committee. |

Note that full information on the approval of the study protocol must also be provided in the manuscript.

## Flow Cytometry

### Plots

Confirm that:

- [x] The axis labels state the marker and fluorochrome used (e.g. CD4-FITC).
- [x] The axis scales are clearly visible. Include numbers along axes only for bottom left plot of group (a 'group' is an analysis of identical markers).
- [x] All plots are contour plots with outliers or pseudocolor plots.
- [x] A numerical value for number of cells or percentage (with statistics) is provided.

### Methodology

| | |
|---|---|
| Sample preparation | 5 µL fresh whole blood with sodium citrate (9:1) anticoagulant was dropped on 10 mg NEMs membrane sample for each group (incubation for 10 min). 100 µL PBS, 2 µL antibody CD61 (the platelet markers) and 2 µL antibody CD62P (the dominant platelet activation markers) were added and bound to the samples then oscillated to fully disperse (incubation for 15 min). The samples were diluted using 200 µL PBS then filtrated the NEMs samples using a filter. The percentage of CD61+ and double CD61+/CD62P+ cells was analyzed by a flow cytometer. |
| Instrument | Flow cytometry is analyzed by a flow cytometer (DxP Athena V2-B4-R2, Wuxi Xiatai Biological Technology Co., Ltd.). |
| Software | Flowjo (version 10) is used to the data analysis of flow cytometry. |
| Cell population abundance | The whole blood was used in the platelet assessment, and no sorting was performed in this study. |
| Gating strategy | Preliminary FSC/SSC gates for the starting platelets population, and the samples were further gated for analysis using the markers of CD61 and CD62P. Gating strategy for all flow cytometry experiments are available in the Supplementary Information and Source Data. |

- [x] Tick this box to confirm that a figure exemplifying the gating strategy is provided in the Supplementary Information.
